# Supplementary figures and images for: Microcephaly-associated protein WDR62 shuttles from the Golgi apparatus to the spindle poles in human neural progenitors
Source: eLife. 2023 Jun 5;12:e81716. doi: 10.7554/eLife.81716 (PMC10241521; doi:10.7554/eLife.81716)

Figure 2 - figure supplement 1 (B)

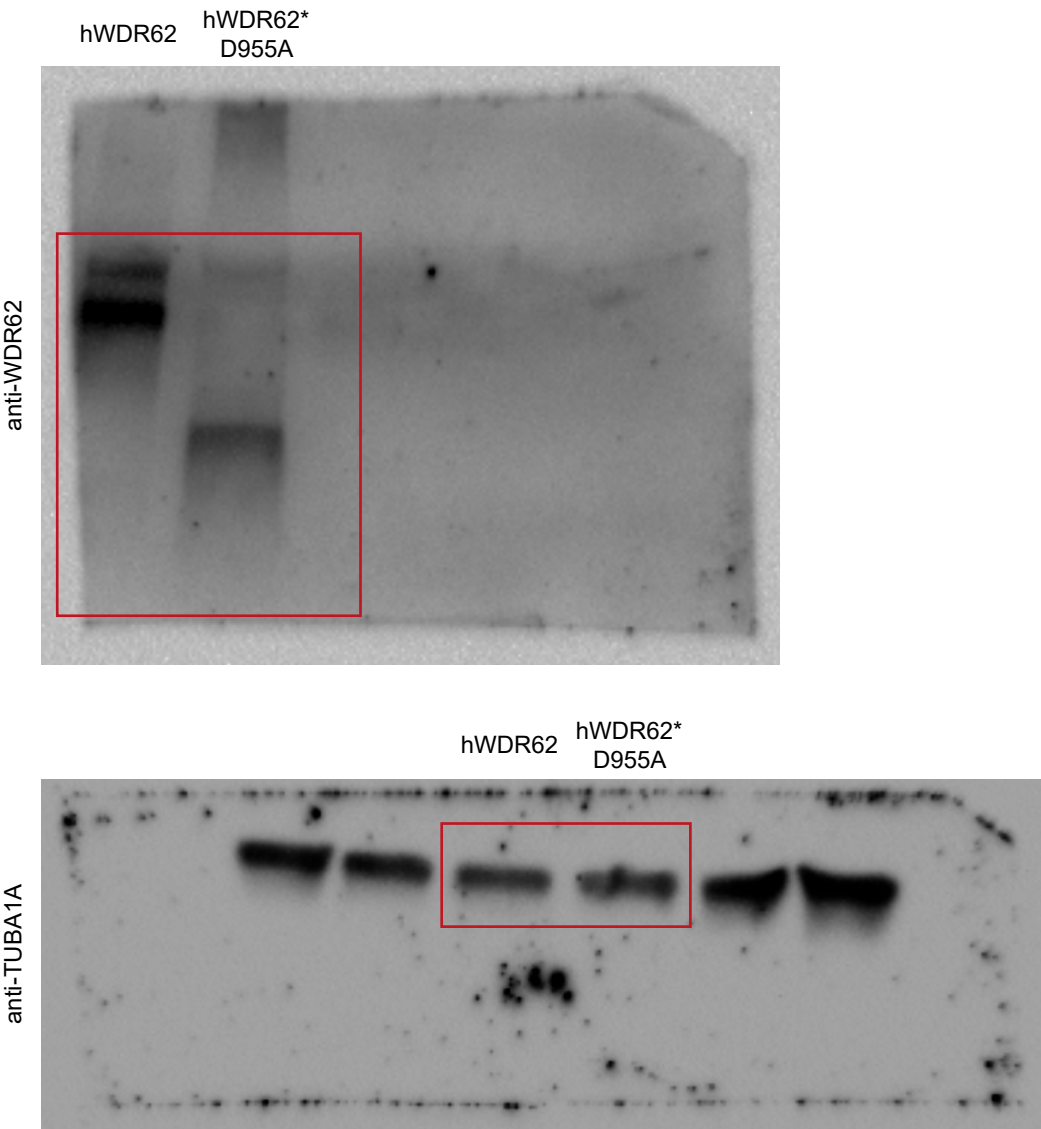

Supplement: Figure 2—figure supplement 2—source data 1. [file elife-81716-fig2-figsupp2-data1.zip › Figure 2-figure supplement 2-source data 1/Blots labelled/Figure 2_figure supplement 2_uncropped labelled.pdf]

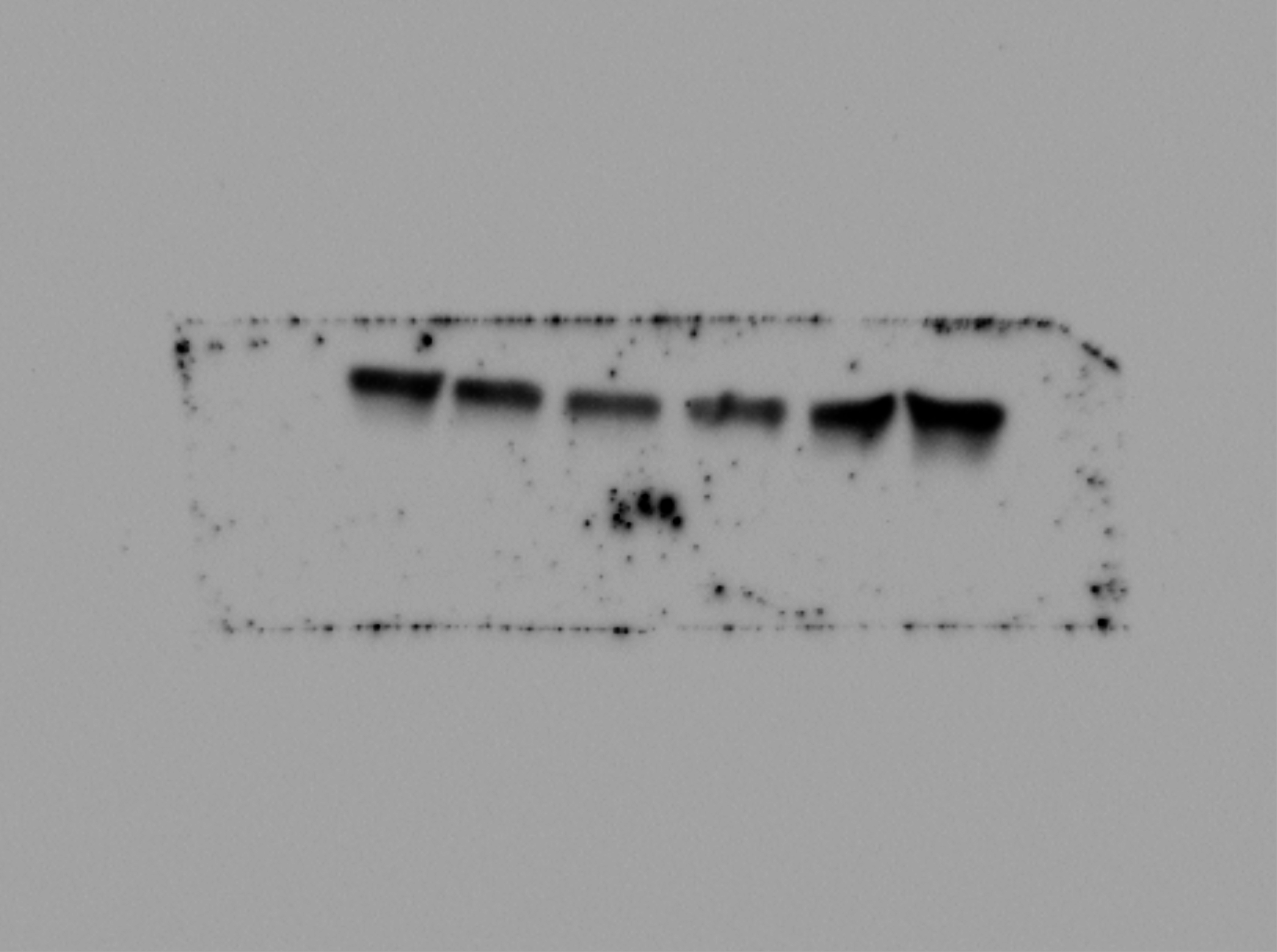

Supplement: Figure 2—figure supplement 2—source data 1. [file elife-81716-fig2-figsupp2-data1.zip › Figure 2-figure supplement 2-source data 1/Raw blots/WB_antiTUBA1A_hWDR62+D955A.tif]

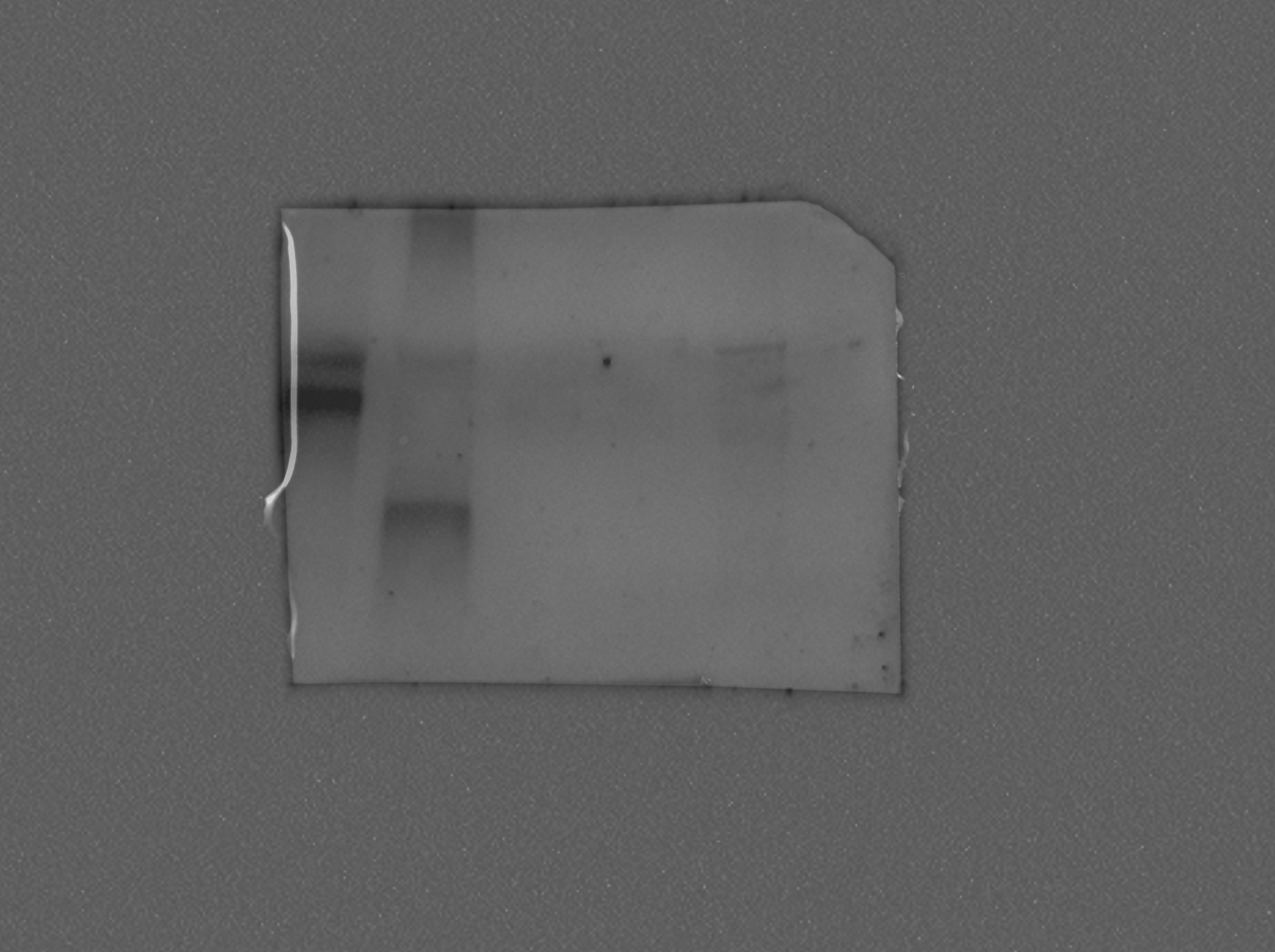

Supplement: Figure 2—figure supplement 2—source data 1. [file elife-81716-fig2-figsupp2-data1.zip › Figure 2-figure supplement 2-source data 1/Raw blots/WB_antiWDR62_hWDR62+D955A composite ladder.tif]

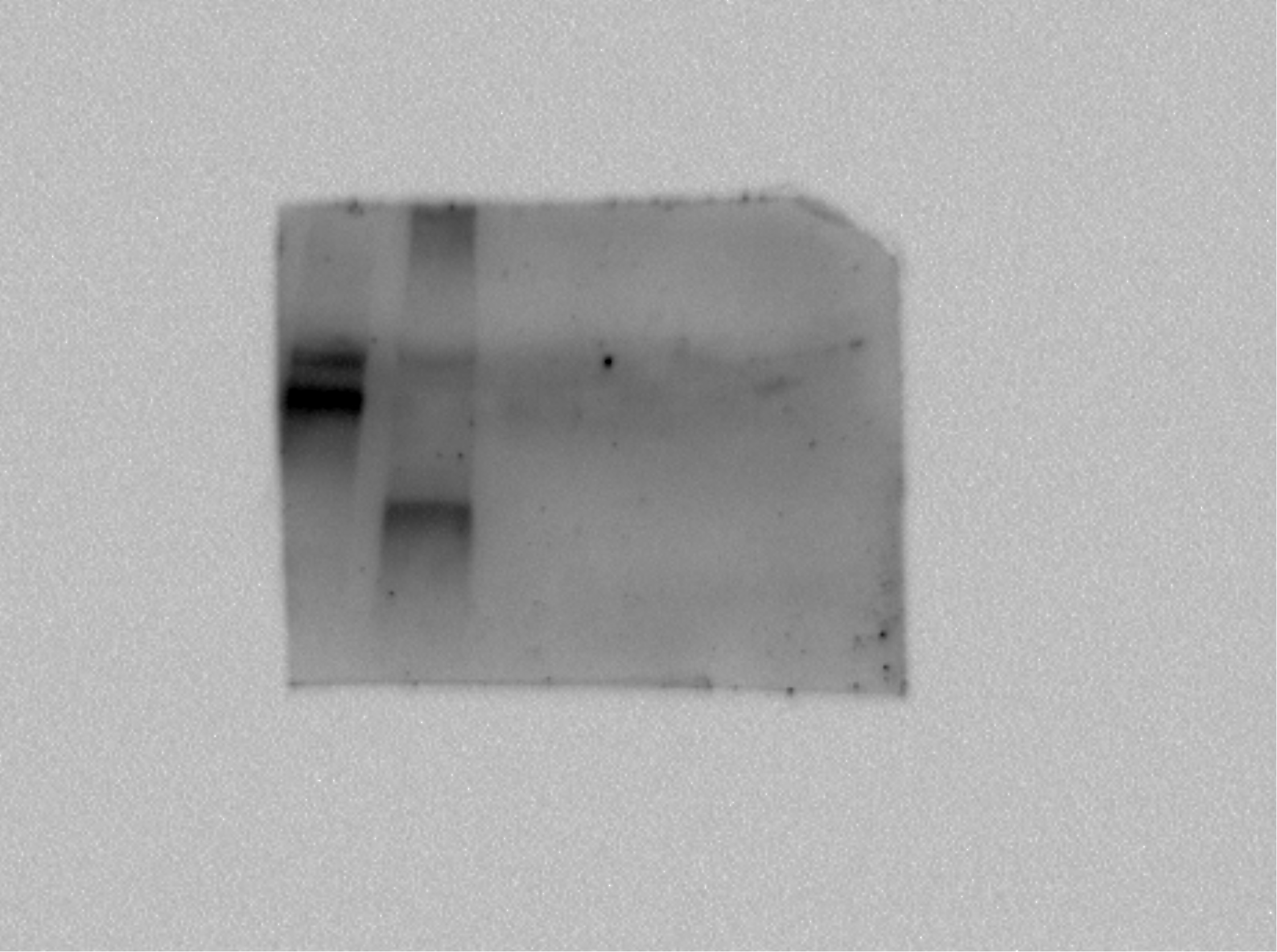

Supplement: Figure 2—figure supplement 2—source data 1. [file elife-81716-fig2-figsupp2-data1.zip › Figure 2-figure supplement 2-source data 1/Raw blots/WB_antiWDR62_hWDR62+D955A.tif]

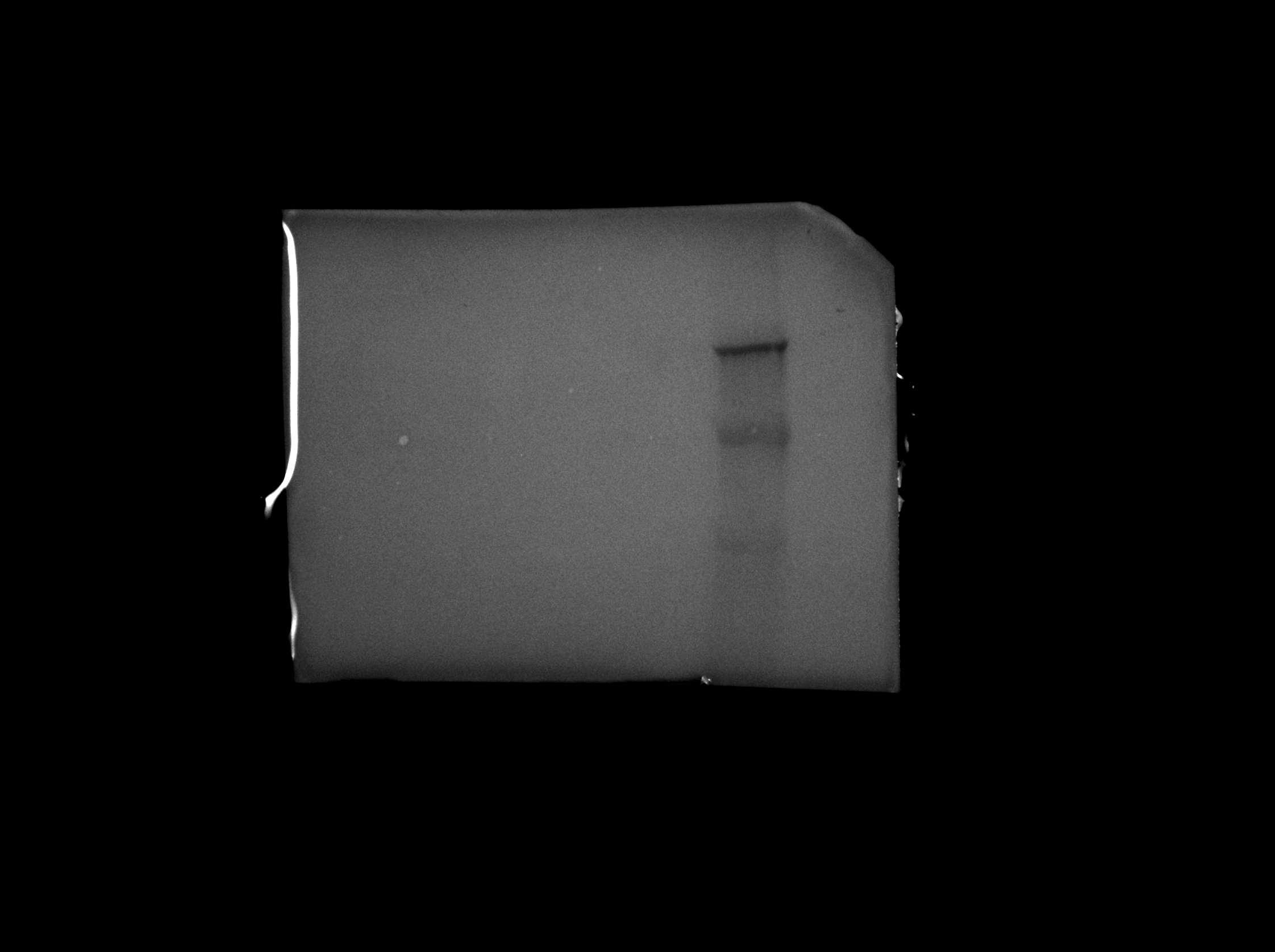

Supplement: Figure 2—figure supplement 2—source data 1. [file elife-81716-fig2-figsupp2-data1.zip › Figure 2-figure supplement 2-source data 1/Raw blots/WB_antiWDR62_ladder.tif]

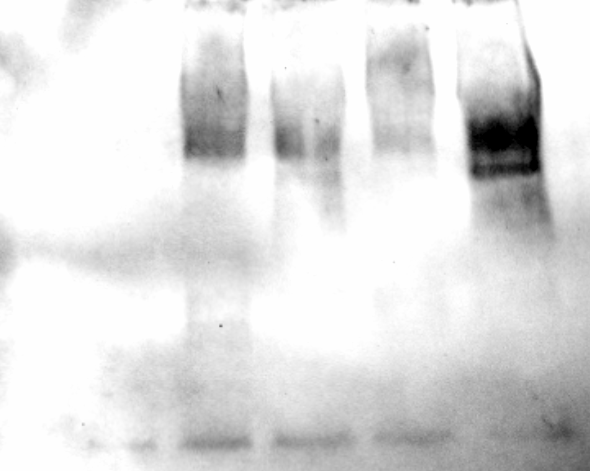

Supplement: Figure 3—figure supplement 1—source data 1. [file elife-81716-fig3-figsupp1-data1.zip › Figure 3-figure supplement 1-source data 1/Raw blots/hWDR62 input FLAG.tif]

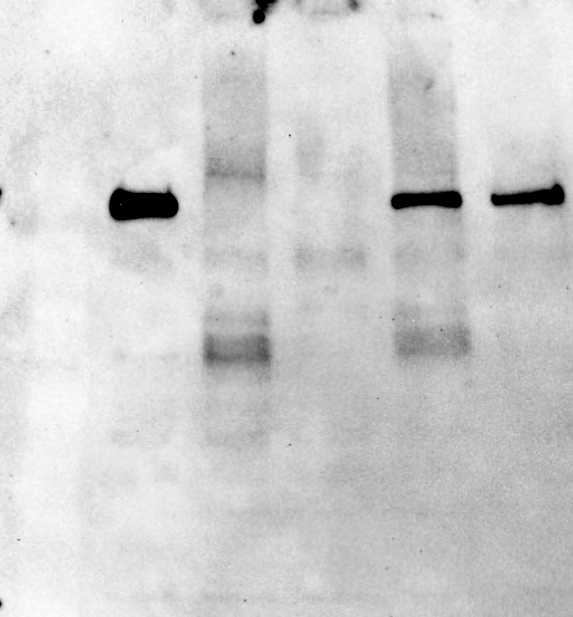

Supplement: Figure 3—figure supplement 1—source data 1. [file elife-81716-fig3-figsupp1-data1.zip › Figure 3-figure supplement 1-source data 1/Raw blots/hCDK5RAP2 input Myc.tif]

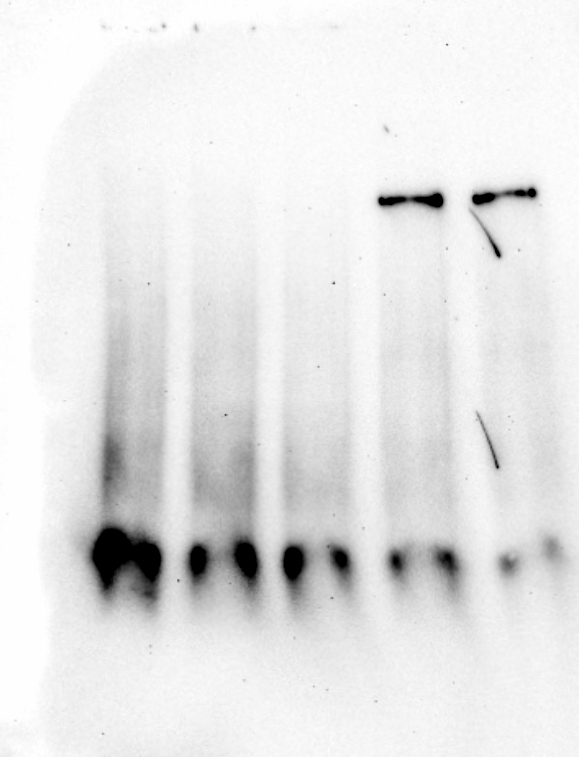

Supplement: Figure 3—figure supplement 1—source data 1. [file elife-81716-fig3-figsupp1-data1.zip › Figure 3-figure supplement 1-source data 1/Raw blots/hCDK5RAP2 IP w_WDR62-FLAG.tif]

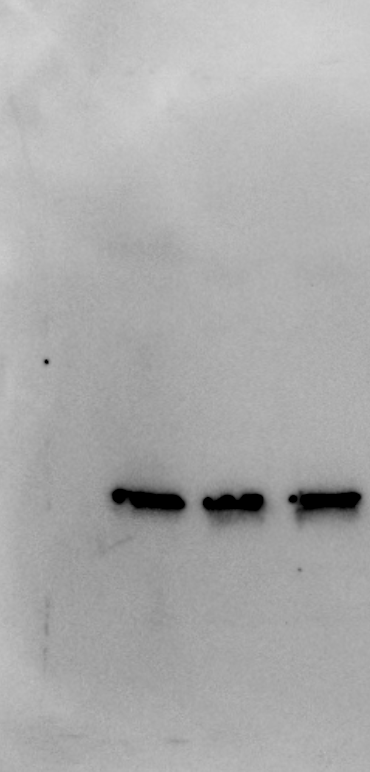

Supplement: Figure 3—figure supplement 1—source data 2. [file elife-81716-fig3-figsupp1-data2.zip › Figure 3-figure supplement 1-source data 2/Raw blots/hAURKA input Myc.tif]

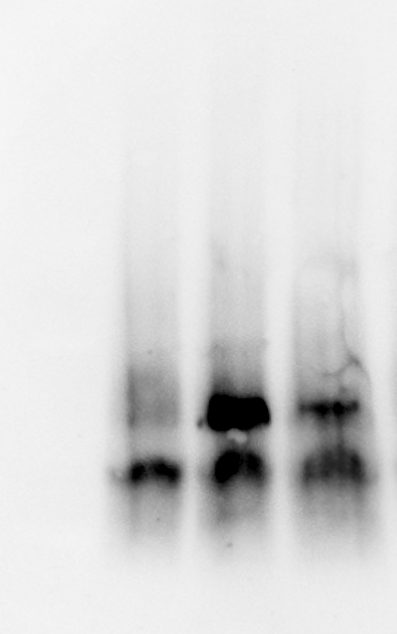

Supplement: Figure 3—figure supplement 1—source data 2. [file elife-81716-fig3-figsupp1-data2.zip › Figure 3-figure supplement 1-source data 2/Raw blots/hAURKA IP w_WDR62-FLAG.tif]

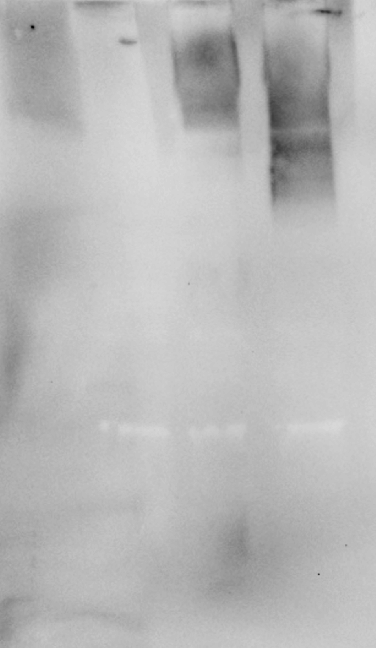

Supplement: Figure 3—figure supplement 1—source data 2. [file elife-81716-fig3-figsupp1-data2.zip › Figure 3-figure supplement 1-source data 2/Raw blots/hWDR62 input FLAG.tif]

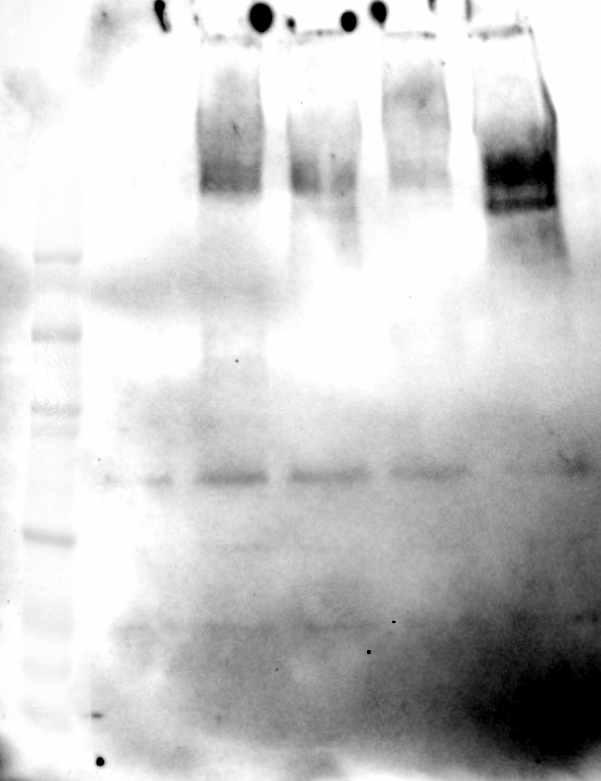

Supplement: Figure 3—figure supplement 1—source data 3. [file elife-81716-fig3-figsupp1-data3.zip › IP images with MW markers/Figure 3 figure supplement 1 Panel E/Myc-CDK5RAP2 Input IB-FLAG with ladder.tif]

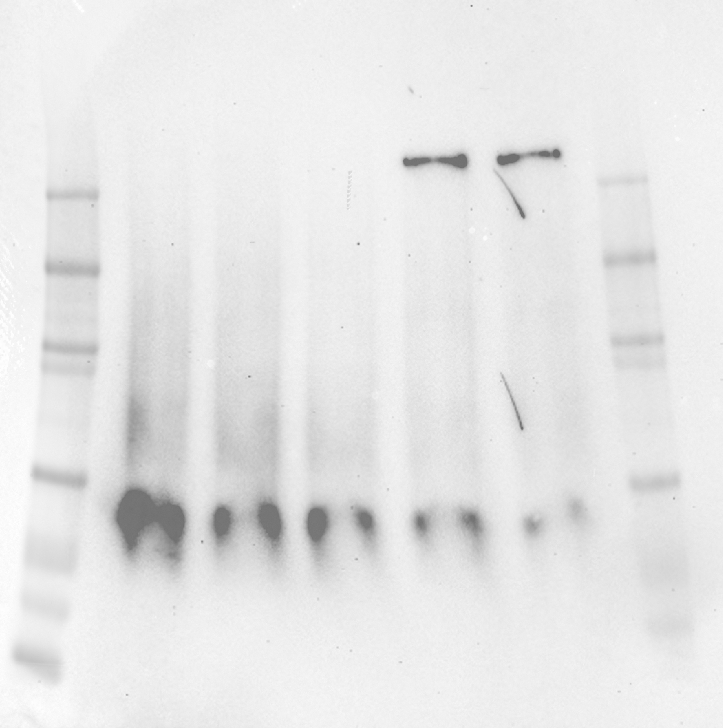

Supplement: Figure 3—figure supplement 1—source data 3. [file elife-81716-fig3-figsupp1-data3.zip › IP images with MW markers/Figure 3 figure supplement 1 Panel E/Myc-CDK5RAP2 IP-FLAG IB-Myc with ladder.tif]

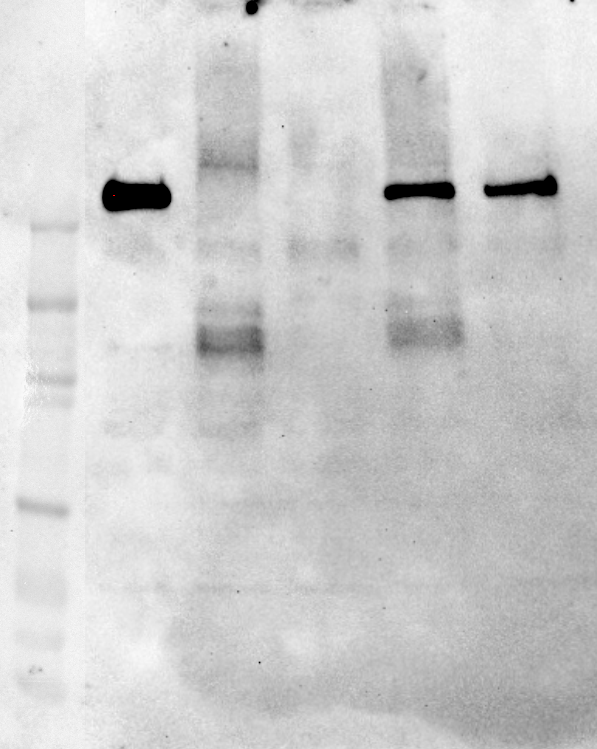

Supplement: Figure 3—figure supplement 1—source data 3. [file elife-81716-fig3-figsupp1-data3.zip › IP images with MW markers/Figure 3 figure supplement 1 Panel E/Myc-CDK5RAP2 Input IB-Myc with ladder.tif]

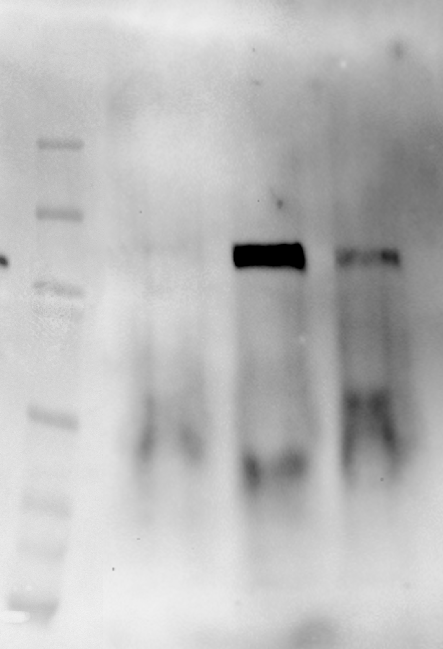

Supplement: Figure 3—figure supplement 1—source data 3. [file elife-81716-fig3-figsupp1-data3.zip › IP images with MW markers/Figure 3 figure supplement 1 Panel G/Myc-TPX2 IP-FLAG IB-Myc with ladder.tif]

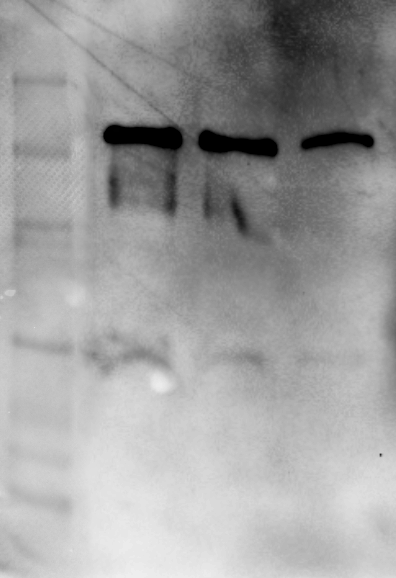

Supplement: Figure 3—figure supplement 1—source data 3. [file elife-81716-fig3-figsupp1-data3.zip › IP images with MW markers/Figure 3 figure supplement 1 Panel G/Myc-TPX2 Input IB-Myc w:ith adder.tif]

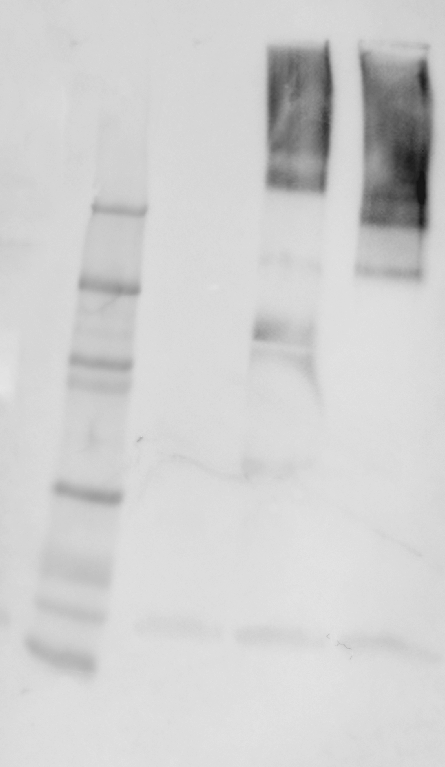

Supplement: Figure 3—figure supplement 1—source data 3. [file elife-81716-fig3-figsupp1-data3.zip › IP images with MW markers/Figure 3 figure supplement 1 Panel G/Myc-TPX2 IP-FLAG IB-FLAG with ladder.tif]

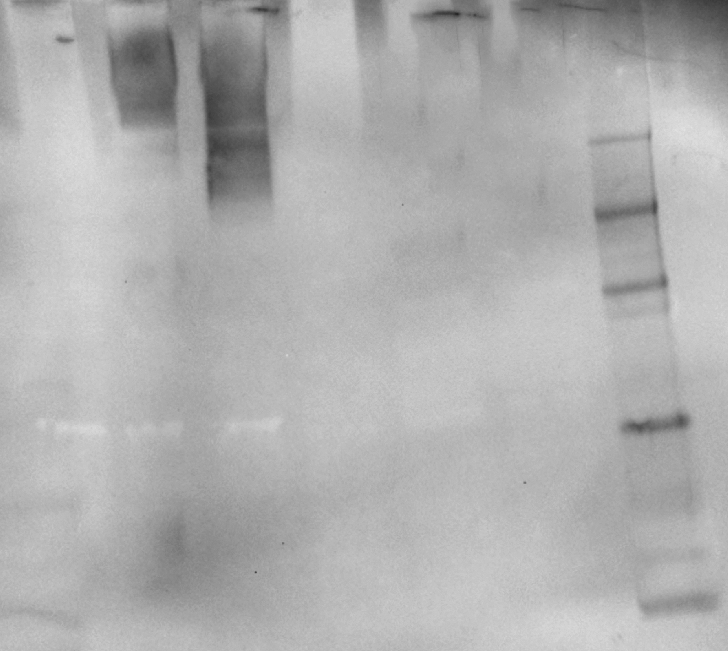

Supplement: Figure 3—figure supplement 1—source data 3. [file elife-81716-fig3-figsupp1-data3.zip › IP images with MW markers/Figure 3 figure supplement 1 Panel F/Myc-AURKA Input IB-FLAG with ladder.tif]

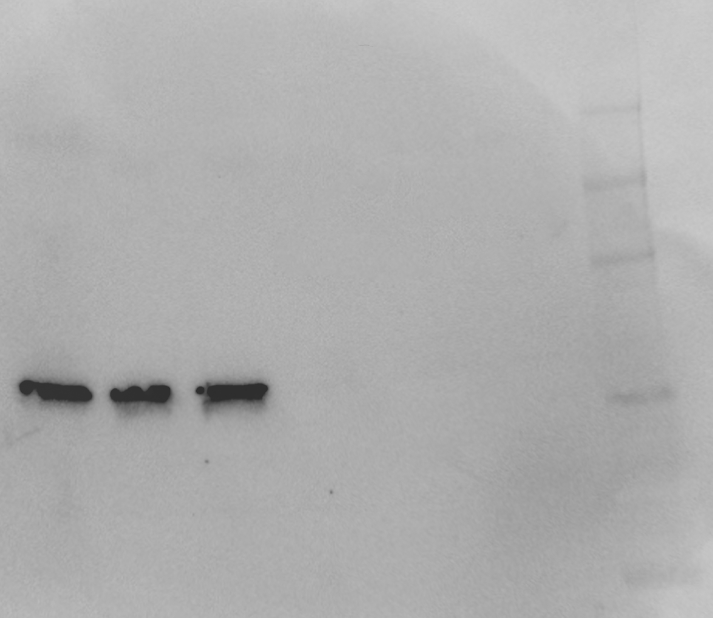

Supplement: Figure 3—figure supplement 1—source data 3. [file elife-81716-fig3-figsupp1-data3.zip › IP images with MW markers/Figure 3 figure supplement 1 Panel F/Myc-AURKA Input IB-Myc with ladder.tif]

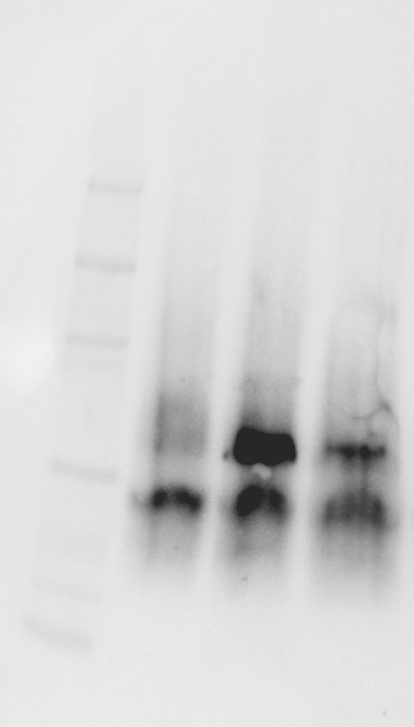

Supplement: Figure 3—figure supplement 1—source data 3. [file elife-81716-fig3-figsupp1-data3.zip › IP images with MW markers/Figure 3 figure supplement 1 Panel F/Myc-AURKA IP-FLAG IB-Myc with ladder.tif]

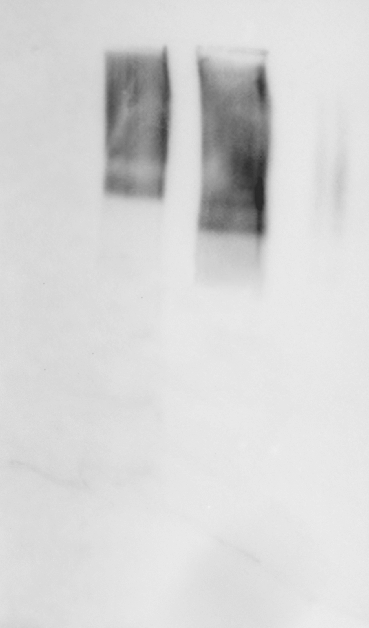

Supplement: Figure 3—figure supplement 1—source data 3. [file elife-81716-fig3-figsupp1-data3.zip › Raw blots/hWDR62 input FLAG.tif]

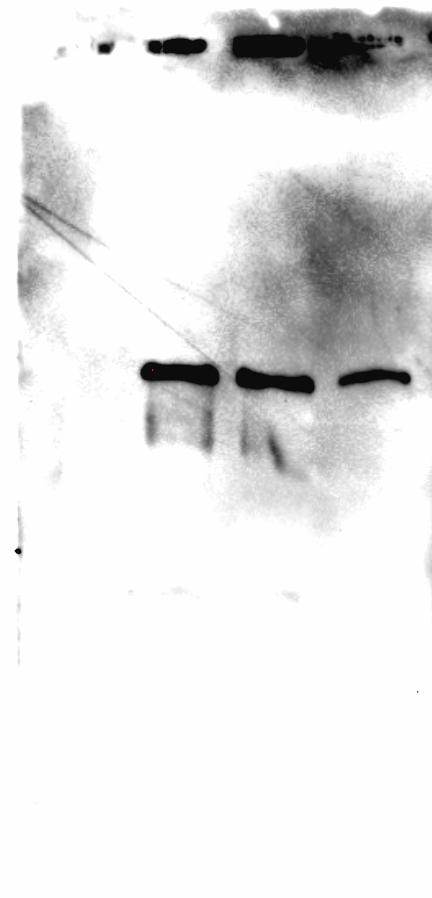

Supplement: Figure 3—figure supplement 1—source data 3. [file elife-81716-fig3-figsupp1-data3.zip › Raw blots/hTPX2 input Myc.tif]

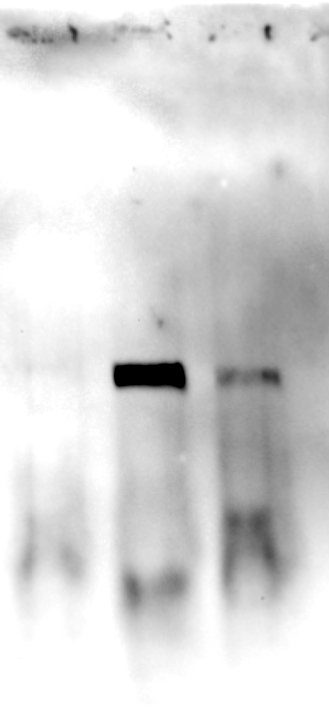

Supplement: Figure 3—figure supplement 1—source data 3. [file elife-81716-fig3-figsupp1-data3.zip › Raw blots/hTPX2 IP w_WDR62-FLAG.tif]
