# Supplementary material for: Microcephaly-associated protein WDR62 shuttles from the Golgi apparatus to the spindle poles in human neural progenitors
Source: eLife. 2023 Jun 5;12:e81716. doi: 10.7554/eLife.81716 (PMC10241521; doi:10.7554/eLife.81716)

Figure 4 - figure supplement 1 (F)

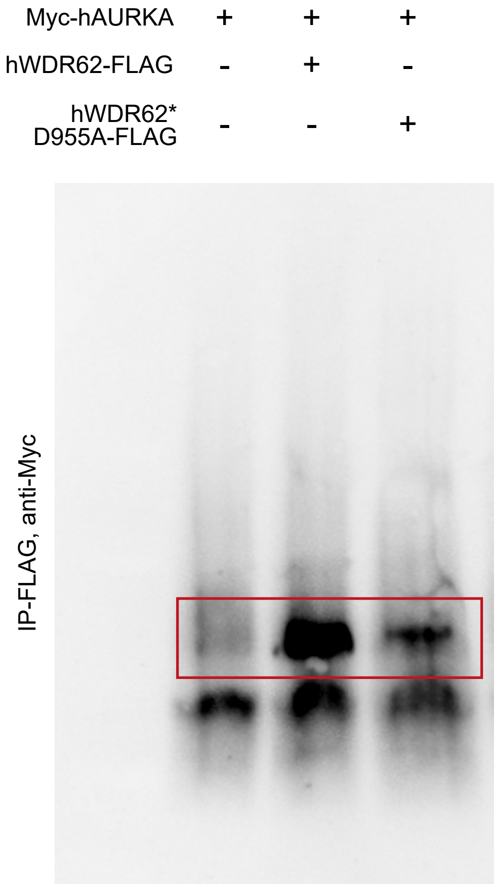

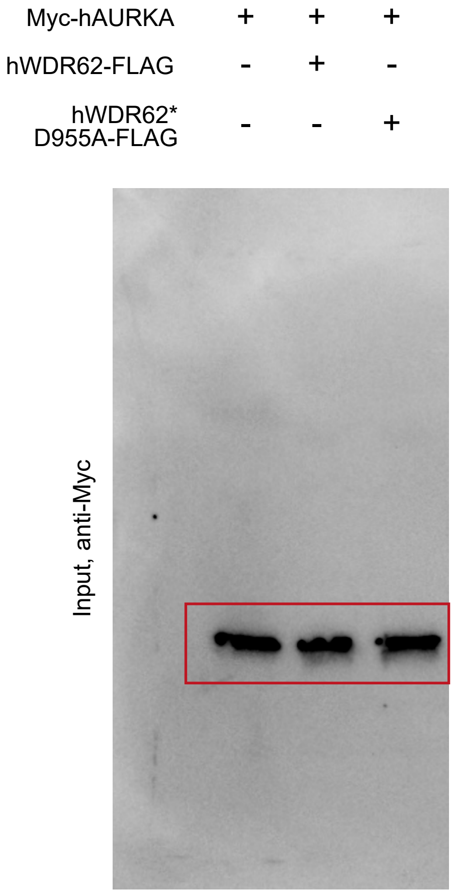

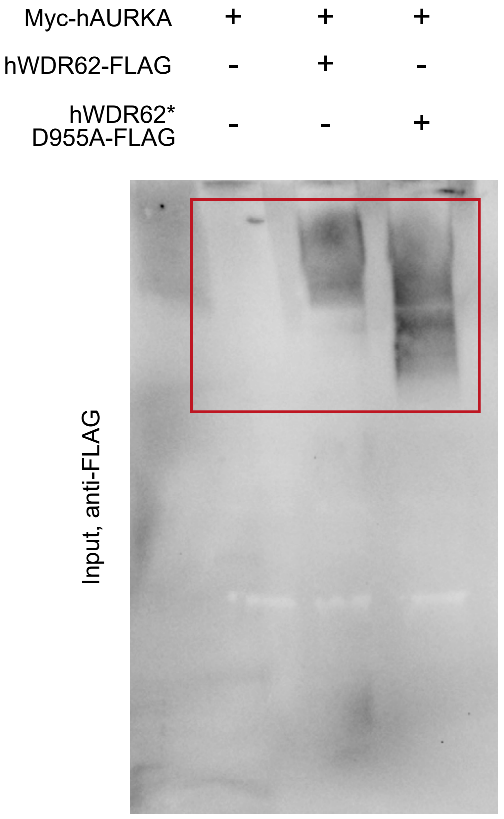

Supplement: Figure 3—figure supplement 1—source data 2. [file elife-81716-fig3-figsupp1-data2.zip › Figure 3-figure supplement 1-source data 2/Blots labelled/Figure 3_figure supplement 1F_uncropped labelled.pdf]
