# Supplementary material for: Microcephaly-associated protein WDR62 shuttles from the Golgi apparatus to the spindle poles in human neural progenitors
Source: eLife. 2023 Jun 5;12:e81716. doi: 10.7554/eLife.81716 (PMC10241521; doi:10.7554/eLife.81716)

Figure 4 - figure supplement 1 (G)

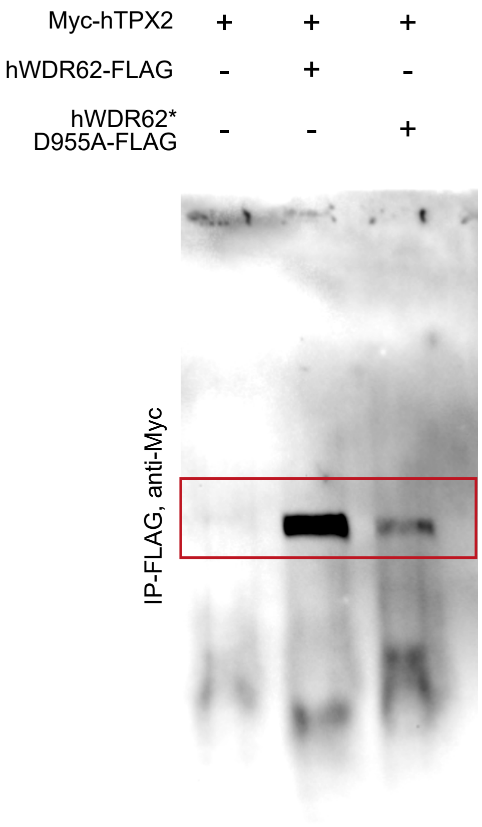

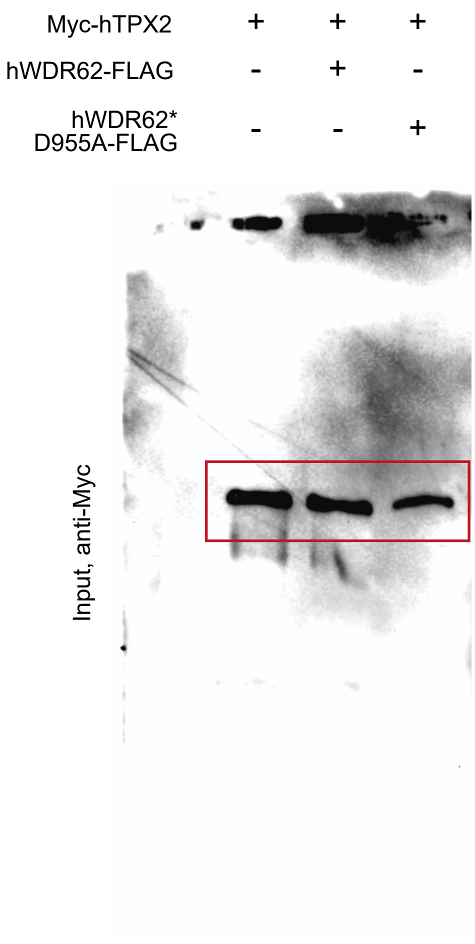

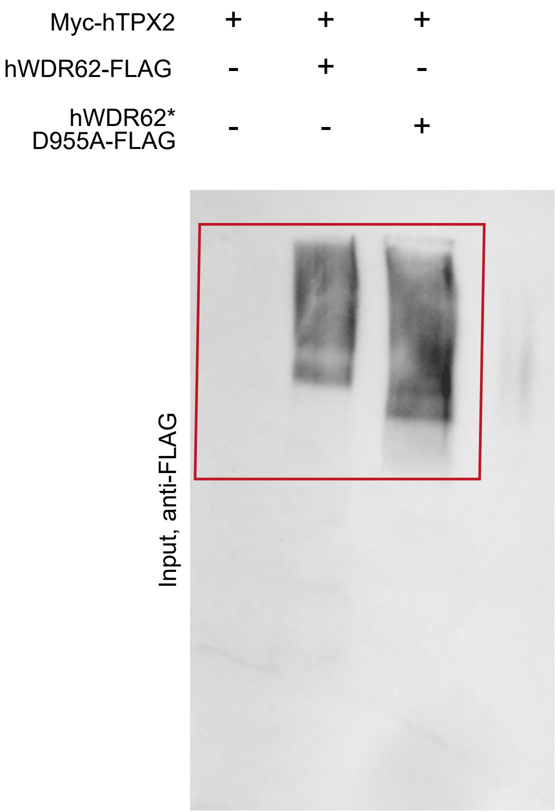

Supplement: Figure 3—figure supplement 1—source data 3. [file elife-81716-fig3-figsupp1-data3.zip › Blots labelled/Figure 3_figure supplement 1G_uncropped labelled.pdf]
